# Case report: Thrombotic microangiopathy in pediatric multisystem inflammatory syndrome associated with COVID-19: a case series

**DOI:** 10.3389/fped.2023.1254308

**Published:** 2023-10-12

**Authors:** Hülya Nalçacıoğlu, H. Gözde Önal, Burcu Bozkaya Yücel, Demet Tekcan Karali, Emine Erdeniz, Gökçen Öz Tuncer, Özlem Aydoğ

**Affiliations:** ^1^ Pediatric Nephrology Department, Ondokuz Mayis University Faculty of Medicine, Samsun, Türkiye; ^2^ Pediatric Rheumatology Department, Ondokuz Mayis University Faculty of Medicine, Samsun, Türkiye; ^3^ Pediatric Infectious Department, Ondokuz Mayis University Faculty of Medicine, Samsun, Türkiye; ^4^ Ondokuz Mayis University Faculty of Medicine Pediatric Neurology Department, Samsun, Turkey; ^5^ Pediatric Nephrology- Rheumatology Department, Ondokuz Mayis University Faculty of Medicine, Samsun, Türkiye

**Keywords:** COVID-19, multisystem inflammatory syndrome, thrombotic microangiopathy, IVIG, anakinra, eculizimab

## Abstract

**Introduction:**

This report provides insight into three distinct pediatric cases exhibiting a nexus between multisystem inflammatory syndrome in children (MIS-C) and thrombotic microangiopathy (TMA) triggered by COVID-19. The aim is to underscore the range of clinical presentations and the essentiality of early interventions.

**Case presentations:**

This report presents three cases aged 10 months, 7 years, and 3 years with persistent fever, diarrhea, nausea, and vomiting. The first case, a 10-month-old girl, demonstrated acute kidney injury (AKI) and microangiopathic hemolytic anemia (MAHA) following a COVID-19 infection. Despite initial negative SARS-CoV-2 RT-PCR results, her condition escalated rapidly, presenting increased levels of LDH (peaking at 4,200 U/L) and requiring renal replacement therapy (RRT) to manage deteriorating renal function. Interventions with eculizumab and anakinra led to marked improvements, with a stable follow-up of 13 months post-hospitalization. The second case involved a 7-year-old girl who developed symptoms of MIS-C, hemolytic uremic syndrome (HUS), and posterior reversible encephalopathy syndrome (PRES) post-exposure to COVID-19, evidenced by heightened LDH levels (3,522 U/L at peak). After a precarious period of deteriorating kidney function and exacerbated hypertension, she responded positively to treatments, inclusive of IVIG, steroid therapies, and eculizumab, with a favorable 6-month follow-up showcasing stable laboratory results. The third case discusses a 3-year-old boy, without any medical history, manifesting HUS symptoms and COVID-19 infection. He exhibited increased LDH levels (peaking at 3,946 U/L) alongside elevated creatinine, marking renal impairment. He responded well to hemodialysis, IVIG, and steroid therapy, showcasing substantial recovery by the 19th day of hospitalization, which marked his discharge with a tapering steroid regimen.

**Conclusion:**

This case series underscores that MIS-C-associated TMA is a significant complication in pediatric COVID-19. Our findings illuminate the potential for treatment success but simultaneously emphasize the need for a more comprehensive understanding of the underlying pathophysiology.

## Introduction

Severe acute respiratory syndrome coronavirus 2 (SARS-CoV-2) infection, commonly known as coronavirus disease 2019 (COVID-19), primarily affects the respiratory system and can cause acute respiratory distress syndrome in severe cases, particularly among older individuals with comorbidities (1–3). However, children generally experience milder symptoms or are even asymptomatic when infected with SARS-CoV-2 (2–4).

Multisystem inflammatory syndrome in children (MIS-C) has emerged as a significant cause of hospitalization in children associated with SARS-CoV-2 infection. MIS-C is characterized by fever, inflammation, and organ dysfunction, often occurring several weeks after a COVID-19 infection (4–7). It has been observed that MIS-C is frequently accompanied by acute kidney injury (AKI), with reported incidence ranging from 10% to 60% of cases (5, 8, 9).

Thrombotic microangiopathy (TMA) is a potential complication of COVID-19-related hyperinflammatory syndrome and is reported in adults and the pediatric population. Complement activation, specifically the alternative pathway, has been implicated in the pathogenesis of TMA in the context of SARS-CoV-2 infection (10). Several studies have highlighted the occurrence of TMA in pediatric patients with MIS-C and SARS-CoV-2 infection (11–15). Recognizing and promptly treating TMA in children with MIS-C and SARS-CoV-2 infection is essential, as it can be associated with significant morbidity and mortality. Herein, we presented three patients diagnosed with MIS-C and TMA related to COVID-19.

## Case reports

### Case 1

The first patient was a 10-month-old girl with no previous medical history or family history of illness. She was admitted to the hospital with a 1-day history of diarrhea, vomiting, and a 3-day history of fever. Upon admission, her vital signs were as follows: fever of 38.7°C, heart rate of 140 bpm, blood pressure of 95/55 mm Hg, respiratory rate of 30 bpm, and oxygen saturation (SpO2) of 98% on room air. Physical examination revealed abdominal tenderness but was otherwise normal.

Initial laboratory results showed signs of inflammation, including elevated levels of C-reactive protein (CRP) at 147 mg/L (normal <5 mg/L) and hyperferritinemia at 1,075 ng/ml. The patient also had an increased white blood cell count of 20,490 per mm^3^ and high D-dimer, LDH, AST, and ALT levels. Additionally, she had a low platelet count of 48,000 per mm^3^. Electrolytes and renal function were normal. Urinalysis revealed 2+ proteinuria. Urine and blood cultures were negative, and there was no evidence of an active infection or neoplasia. Bone marrow examination did not show hemophagocytosis or leukemic infiltration.

The SARS-CoV-2 reverse transcriptase-polymerase chain reaction (RT-PCR) test from a nasopharyngeal swab returned negative; the COVID-19 serology test was positive for IgG (>250 UA/ml) and negative for IgM. Echocardiography showed normal findings, while abdominal ultrasound revealed colitis findings and increased renal parenchymal echogenicity. The patient was diagnosed with MIS-C and treated with intravenous immunoglobulin (IVIG) at 2 g/kg/day and methylprednisolone at 10 mg/kg/day for a 3-day therapy. She continued with a maintenance dose of prednisolone at 2 mg/kg daily. Empiric broad-spectrum antibiotics were also initiated.

On the fourth day of her illness, the patient experienced a decrease in urine output, edema, anemia, thrombocytopenia, and acute kidney injury (AKI). Laboratory results showed a high creatinine level (1.8 mg/dl), low haptoglobin level, reticulocytosis (8%), and an LDH level of 4,200 U/L. The direct Coombs test was negative, and a peripheral blood smear examination revealed schistocytes. ADAMTS-13 activity and levels were within the normal range, while complement analysis showed decreased complement 3 levels. On the fifth day of hospitalization, she was transferred to the pediatric intensive care unit (PICU) for renal replacement therapy, including peritoneal dialysis. It was suspected that the patient developed microangiopathic hemolytic anemia (MAHA) due to MIS-C, as no other explanation could be found. MAHA associated with MIS-C, the patient received eculizumab treatment to reduce microangiopathic frequency. Her symptoms began to improve, with a decrease in ferritin levels, normalization of LDH levels, and stabilization of platelet and hemoglobin levels. The patient was transferred to the nephrology wards on hospital day 15 and while on stable under peritoneal dialysis. The urine output increased, and peritoneal dialysis was stopped. However, despite improved kidney function, the patient continued to have persistently high levels of ferritin and LDH and a declining platelet count. Anakinra was added to the treatment regimen after the second dose of eculizumab (on the 22nd day of hospitalization). Platelet levels increased, and ferritin levels decreased during the first week of anakinra treatment. In the outpatient follow-up, anakinra was eventually stopped after 2 months. Steroid therapy was discontinued at the end of the fourth month. The patient has since been doing well, with a follow-up period of 13 months.

### Case 2

The second patient was a 7-year-old girl admitted to the hospital with complaints of nausea, vomiting, bloody diarrhea, and fever, which had been present for 2 days. She reported swimming in polluted lake water 1 week before the onset of symptoms.

On examination, the patient appeared ill but not in distress. She was febrile, with a blood pressure of 110/70 mmHg, a pulse rate of 85 bpm, a respiratory rate of 26 bpm, and an oxygen saturation of 98%.

Investigations revealed impaired renal function (BUN 70 mg/dl, serum creatinine 1.4 mg/dl), elevated LDH (3,522 U/L), ferritin (1,776 ng/ml), CRP levels (104 mg/dl), and decreased haptoglobin, hemoglobin, and platelet levels (<0.29 g/L, 7.5 g/dl, 16,000/µl, respectively). Peripheral blood smear examination showed the presence of schistocytes, indicating hemolysis. Complement evaluation revealed normal ADAMTS13 activity but decreased serum C3 level. The occult blood test was positive, and PCR for SARS-CoV-2 infection was negative, but COVID-19 total antibody testing was positive for IgG (>250 UA/ml). The stool PCR for Shiga toxin was negative.

During the follow-up period, the patient's urine output decreased, and her kidney function deteriorated, with an increased creatinine level reaching 4.5 mg/dl, accompanied by worsened blood pressure control. Hemodialysis was initiated, but the patient continued to have persistent fever and elevated CRP and ferritin levels. MIS-C and hemolytic uremic syndrome (HUS) diagnoses were considered due to persistent MAHA, thrombocytopenia, decreased haptoglobin levels, increased inflammation markers, and positive COVID-19 antibodies. IVIG and pulse steroid therapy (10 mg/kg/day for 3 days) was initiated then oral prednisolone treatment was started at 2 mg/kg/day.

On the 8th day of hospitalization, the patient developed seizures, loss of consciousness, facial palsy, and hypertension. She was transferred to the PICU for the neurologic complication and hypertension. Brain MRI findings were consistent with posterior reversible encephalopathy syndrome (PRES), and no signs of papilledema were observed during the eye consultation. Electroencephalography (EEG) did not show epileptiform activity. Eculizumab treatment was administered, followed by two plasmapheresis sessions. The patient was transferred to the nephrology clinic and the second dose of eculizumab was given 1 week later. Hypertension was managed with antihypertensive medications (amlodipine, furosemide, captopril, propranolol), and hemodialysis treatments were continued. Acute phase reactants showed improvement following IVIG and steroid treatments. Kidney function improved, and hemodialysis was discontinued on the 20th day of hospitalization. The patient was discharged with amlodipine and steroids on the 26th day of hospitalization. Laboratory values at discharge showed serum creatinine of 0.78 mg/dl, LDH of 482 U/L, ferritin of 1,370 ng/ml, hemoglobin of 9.6 g/dl, and platelet count of 519,000/µl. Prednisolone treatment was prescribed at 10 mg (0.4 mg/kg) every other day and continued with a tapering regimen before discontinuation. The patient remained clinically stable with favorable laboratory results during a 6-month follow-up.

### Case 3

A 3-year-old male patient complained of bloody diarrhea, vomiting, fever, and decreased urine output for 1 week. He had no known medical conditions and no previous hospitalizations. His parents had no consanguinity and no family history of kidney disease.

During the physical examination upon admission to the hospital, the patient had a high fever of 38.1°C. His blood pressure was 110/70 mmHg, heart rate was 140 beats per minute, respiratory rate was 28 breaths per minute, and oxygen saturation was 99%. Edema was noted in his eyelids and scrotum.

Initial laboratory results at admission revealed hyponatremia (sodium: 128 mg/dl), elevated blood urea nitrogen (BUN: 52 mg/dl), increased creatinine (Cr: 4.03 mg/dl), elevated lactate dehydrogenase (LDH: 3,946 U/L), elevated ferritin (1,468 ng/ml), hemoglobin (Hb: 10.8 g/dl), thrombocytopenia (platelets: 43,000/µl), and elevated d-dimer (>10,000 ng/ml). C3 was low, while C4 was within the normal range. ADAMTS13 activity (84%) fell within the normal reference range (40%–130%). The patient tested positive for COVID-19 through PCR and total antibody (23.5 U/ml) testing. Peripheral blood smear analysis revealed the presence of schistocytes and other signs of hemolysis. Shiga toxin-mediated HUS was ruled out by the absence of Shiga toxin in the stool PCR.

Abdominal ultrasound showed small bowel loops enlargement, minimal pelvis-free fluid, and grade 2 increased echogenicity in the renal parenchyma. Hemodialysis was initiated after 12 h of anuria since hospitalization. Along with renal failure, the patient's thrombocytopenia, anemia, and elevated levels of AST, ALT, LDH, ferritin, and d-dimer led to a diagnosis of MIS-C and hemolytic uremic syndrome.

IVIG treatment (2 g/kg) was administered on the first day of hospitalization. On the sixth day, the patient exhibited an upward gaze in his eyes. He was transferred to PICU for the management of seizure. The seizure resolved spontaneously and did not recur. EEG and cranial MRI results were normal. Pulse steroid therapy was initiated on the sixth day at a dose of 15 mg/kg/day due to a lack of improvement in laboratory findings and worsening clinic conditions. Pulse steroid therapy was given for 3 days, followed by continued steroid treatment at 2 mg/kg. The patient improved clinical and laboratory findings with IVIG, steroids, and supportive therapies. Renal function and urine output improved, and hemodialysis treatment was discontinued on the 12th day of hospitalization.

At discharge, blood tests showed BUN of 41 mg/dl, Cr of 0.64 mg/dl, LDH of 580 U/L, ferritin of 968 ng/ml, CRP of 0.6 mg/dl, Hb of 8.1 g/dl, and platelets of 213,000/µl. The patient was discharged on the 19th day of hospitalization with a tapering steroid therapy regimen, requiring no other post-discharge care. Steroid therapy was gradually tapered and discontinued over 8 weeks. Table 1 summarizes the patient's clinical laboratory -treatment and outcome.

**Table 1:** Summary of the patient's clinical laboratory -treatment and outcome.

	Case 1	Case 2	Case 3
Age	10-month-old girl	7-year-old girl	3-year-old boy
Symptoms	Diarrhea, vomiting, fever, abdominal pain	Nausea, vomiting, bloody diarrhea, fever	Bloody diarrhea, vomiting, fever, decreased urine output
SARS-CoV-2 PCR	Negative	Negative	Positive
COVID-19 serology	Positive IgG (>250 UA/ml)	Positive IgG (>250 UA/ml)	Positive IgG (23.5 U/ml)
Laboratory findings (admission)			
Hg (g/dl)	11.4	7.5	10.8
Thrombocyte/µl	48,000	16,000	43,000
Leukocyte/mm^3^	20,490	14,700	12,600
CRP (mg/L) (5 < N)	147	104	68
D-dimer (ng/ml)	>10,000	8,620	>10,000
Fibrinogen (mg/dl) (*n* > 250)	99	178	185
Ferritin (µg/L)	1,075	1,776	1,468
LDH (U/L)	1,526	3,522	3,946
Creatinine (mg/dl)	0.49	1.4	4
Complement C3 g/L (*N* > 0.8)	0.59	0.72	0.64
Treatment (day of hospitalization)	IVIG (day of 2) Pulse methylprednisolone (day of 3–6) Peritoneal dialysis (days of 5–18) Eculizumab (day of 5) Anakinra (day of 22–2 months) Oral steroid (day of 7–4 month)	Hemodialysis (day of 2–20) IVIG (day of 3) Pulse methylprednisolone (day of 4–7) Oral steroid (day of 8–26)	Hemodialysis (day of 1–12) IVIG (day of 1) Pulse methylprednisolone (day of 6–9) Oral steroid (day of 9–2 months)
Recovery or noteworthy notes-	Renal recovery (day of 18) Peritoneal dialysis was discontinued Exacerbation of MIS-C (day of 22) Hematologic response Improvement of MIS-C (day of 29)	Seizure (day of 8) PRES Plasmapheresis (day of 8–9) Eculizumab (day of 10, and 17) Hematologic response (day of 17)	Seizure (day of 6)
Current status	Recovery (follow-up period of 13 months)	Recovery (follow-up period of 4 months)	Recovery (day of 20)

## Discussion

Herein, we presented three cases highlighting the association between MIS-C and TMA in the context of COVID-19 infection. We discern distinctive yet interconnected features that shed light on the evolving understanding of MIS-C associated with COVID-19. Patient one, a 10-month-old infant, manifested a severe course of the syndrome, characterized by significant laboratory abnormalities, renal involvement requiring dialysis, and a lengthy recovery period marked by integrating various therapeutic approaches, including eculizumab and anakinra. Patient two, a 7-year-old with an exposure history to polluted lake water, experienced both renal and neurological complications, illustrating the multi-faceted nature of MIS-C. Notably, this case demonstrated the occurrence of PRES, a rare but significant neurological manifestation. The third patient, a 3-year-old male, portrayed the MIS-C's severe renal and hematological manifestations, warranting timely interventions such as hemodialysis and pulse steroid therapy.

Severe acute respiratory syndrome coronavirus 2 (SARS-CoV-2) infection, commonly known as COVID-19, primarily affects the respiratory system but can also lead to multisystem involvement in some cases, particularly in children. MIS-C has emerged as a significant cause of hospitalization in children with SARS-CoV-2 infection (1–3). According to the Center for Disease Control and Prevention (CDC), MIS-C presents with persistent fever >38.5°C, rash, conjunctivitis, peripheral edema, severe abdominal pain, and diarrhea. Notably, severe illness requiring hospitalization for clinical symptoms and evidence of COVID-19 exposure within 4 weeks are important factors not present in other case definitions (4, 16, 17).

One of the notable complications of MIS-C is the development of TMA, which involves endothelial dysfunction, microthrombi formation, and multiorgan involvement, including the kidneys (4–7). TMA diagnosis was supported by schistocytes in peripheral blood smears, indicating ongoing hemolysis and laboratory findings such as elevated LDH and decreased haptoglobin levels. Significantly, ADAMTS-13 activity, typically reduced in thrombotic thrombocytopenic purpura, was within the normal range in these cases, distinguishing them from primary TTP. However, complement analysis revealed decreased complement 3 levels in all cases, suggesting complement dysregulation and potential involvement of the alternative pathway in the pathogenesis of TMA.

The pathogenesis of TMA in the context of MIS-C and COVID-19 is not yet fully understood. However, several mechanisms have been proposed based on the existing literature. It has been suggested that the dysregulated immune response triggered by SARS-CoV-2 infection leads to an excessive release of pro-inflammatory cytokines and activation of the complement system. Complement dysregulation, particularly involving the alternative pathway, has been implicated in the development of TMA in MIS-C (4–6). In MIS-C-associated TMA, endothelial cell dysfunction, platelet activation, and the formation of microthrombi in the microvasculature contribute to tissue damage and organ dysfunction. This microangiopathic process can affect various organs, including the kidneys, leading to AKI and renal dysfunction. The underlying endothelial injury and thrombotic microangiopathy in the renal vasculature can result in proteinuria, hematuria, and decreased glomerular filtration rate (7, 8).

Several studies have reported cases of TMA in children with MIS-C and SARS-CoV-2 infection. For example, a study by Diorio et al. (13) described seven children with MIS-C and TMA. These patients exhibited features of TMA, including microangiopathic hemolytic anemia (MAHA), thrombocytopenia, and evidence of endothelial injury. They also demonstrated evidence of complement activation, with low complement levels and increased levels of complement activation products.

The exact mechanisms underlying complement dysregulation and TMA in the context of SARS-CoV-2 infection are not fully understood. However, it has been proposed that the virus can directly activate the complement system, leading to excessive complement activation and subsequent endothelial damage and thrombosis. Genetic factors and host immune responses may also influence the development of complement-mediated TMA in these patients (11, 12, 16, 18).

Recognizing and promptly treating TMA in children with MIS-C and SARS-CoV-2 infection is important, as it can be associated with significant morbidity and mortality. Treatment strategies may include supportive care, immunomodulatory therapies (such as corticosteroids and intravenous immunoglobulin), and complement-targeted therapies (such as eculizumab) (10, 18).

The primary goal of treatment is to control the inflammatory response, prevent further endothelial damage, and mitigate thrombotic complications. Immunomodulatory therapies play a key role in the management of MIS-C and TMA. IVIG is often the first-line therapy in MIS-C due to its anti-inflammatory and immunomodulatory effects. IVIG has been shown to improve clinical outcomes and reduce the risk of coronary artery abnormalities in MIS-C (1, 2, 9). In addition to IVIG, corticosteroids are frequently used to manage MIS-C-associated TMA. Steroids have potent anti-inflammatory properties and can modulate the immune response, thereby reducing endothelial damage and thrombotic complications. The optimal dosing and duration of steroid therapy in MIS-C and TMA remain areas of ongoing investigation (2, 9). Complement inhibitors, such as eculizumab, have shown promise in treating TMA associated with MIS-C and COVID-19. Eculizumab targets the complement cascade, explicitly inhibiting C5, and has successfully managed TMA in other contexts, such as atypical hemolytic uremic syndrome. However, further research is needed to determine the optimal timing, dosing, and duration of eculizumab therapy in MIS-C-associated TMA (10, 11).

There hasn't been a study that directly compares how patients with mild symptoms, monitored with or without immunomodulatory treatments, fare. This means there isn't a one-size-fits-all treatment approach yet. Patients with increasing inflammation markers or high BNP and troponin levels should be hospitalized and watched closely. Given the risks, like cardiac-related complications in patients with high D-dimer and cardiac markers, it seems wise to consider immunomodulatory treatments. If a patient shows severe, potentially life-threatening signs, starting these treatments immediately without waiting for a full diagnosis is best. The Best Available Treatment Study (BATS), a global research effort, found no significant difference in recovery from MIS-C among groups treated with just IVIG, corticosteroids, or a combination of both. It's worth noting that the BATS included a wider range of MIS-C patients, many of whom were less severely ill. Because of this, a stronger initial treatment might be needed for very ill patients to address cardiac problems quickly. Some other treatments, like anakinra and tocilizumab, have been tested, but we don't have solid evidence yet on their effectiveness. Some hospitals have used anakinra for MIS-C patients who didn't improve with the usual steroid or IVIG treatments (18, 19).

In the first case, the patient presented with fever, gastrointestinal symptoms, and signs of inflammation. Despite a negative SARS-CoV-2 PCR test, the serology test showed positive IgG antibodies, indicating previous infection. The patient fulfilled the diagnostic criteria for MIS-C and was treated with IVIG and methylprednisolone. However, on the fourth day of illness, the patient developed features of TMA, including AKI, thrombocytopenia, and MAHA. Treatment with eculizumab for two doses and anakinra improved clinical symptoms and laboratory abnormalities.

Similarly, the second and third cases presented with symptoms consistent with MIS-C and were found to have laboratory evidence of TMA. The patients exhibited hemolysis, thrombocytopenia, AKI features, elevated inflammatory markers, and positive COVID-19 antibody testing. Treatment with IVIG and steroids was initiated, but in the second case, the patient developed PRES and required eculizumab and plasmapheresis for management. Both patients showed clinical improvement and resolution of kidney injury.

Long-term follow-up is crucial for patients with MIS-C-associated TMA to assess renal recovery, monitor for late complications, and optimize overall outcomes. Ongoing research efforts are needed to elucidate the underlying pathophysiological mechanisms, refine diagnostic criteria, and establish evidence-based guidelines for managing MIS-C-associated TMA. Both patients improved after two doses of eculizumab. Eculizumab was discontinued.

In conclusion, this study provides evidence that MIS-C-associated TMA is a significant complication in children with COVID-19 infection, and appropriate treatment approaches can help improve clinical outcomes. Further research is needed to understand the pathophysiological mechanisms better, refine diagnostic criteria, and establish evidence-based guidelines for managing MIS-C-associated TMA. Additionally, long-term follow-up studies are necessary to assess these patients' long-term outcomes and potential late complications.

## Data Availability

The original contributions presented in the study are included in the article/Supplementary Material, further inquiries can be directed to the corresponding author.
